# An Overview on the Marine Neurotoxin, Saxitoxin: Genetics, Molecular Targets, Methods of Detection and Ecological Functions 

**DOI:** 10.3390/md11040991

**Published:** 2013-03-27

**Authors:** Kathleen D. Cusick, Gary S. Sayler

**Affiliations:** 1 The University of Tennessee Center for Environmental Biotechnology, 676 Dabney Hall, Knoxville, TN 37996, USA; E-Mail: sayler@utk.edu; 2 Department of Microbiology, the University of Tennessee, Knoxville, TN 37996, USA; 3 Department of Ecology and Evolutionary Biology, the University of Tennessee, Knoxville, TN 37996, USA; 4 Oak Ridge National Lab, UT-ORNL Joint Institute of Biological Sciences, Oak Ridge, TN 37831, USA

**Keywords:** neurotoxin, saxitoxin, ion channels, copper transporter, phytoplankton, paralytic shellfish toxin

## Abstract

Marine neurotoxins are natural products produced by phytoplankton and select species of invertebrates and fish. These compounds interact with voltage-gated sodium, potassium and calcium channels and modulate the flux of these ions into various cell types. This review provides a summary of marine neurotoxins, including their structures, molecular targets and pharmacologies. Saxitoxin and its derivatives, collectively referred to as paralytic shellfish toxins (PSTs), are unique among neurotoxins in that they are found in both marine and freshwater environments by organisms inhabiting two kingdoms of life. Prokaryotic cyanobacteria are responsible for PST production in freshwater systems, while eukaryotic dinoflagellates are the main producers in marine waters. Bioaccumulation by filter-feeding bivalves and fish and subsequent transfer through the food web results in the potentially fatal human illnesses, paralytic shellfish poisoning and saxitoxin pufferfish poisoning. These illnesses are a result of saxitoxin’s ability to bind to the voltage-gated sodium channel, blocking the passage of nerve impulses and leading to death via respiratory paralysis. Recent advances in saxitoxin research are discussed, including the molecular biology of toxin synthesis, new protein targets, association with metal-binding motifs and methods of detection. The eco-evolutionary role(s) PSTs may serve for phytoplankton species that produce them are also discussed.

## 1. Marine Toxins Overview

Marine neurotoxins are natural products produced primarily by phytoplankton (dinoflagellates and diatoms) along with several types of invertebrates and select species of fish. Their classification as neurotoxins stems from their ability to interact with voltage-gated sodium, potassium and calcium channels and modulate the flux of these ions into various cell types. The neurotoxins produced by phytoplankton, hereafter referred to as algal toxins, encompass a range of molecular structures, from the relatively simple alkaloids and amino acids, to the polyketides, a class of highly diverse compounds in terms of both their structure and biological activity (Table 1). For example, the longest and second-longest continuous carbon atom chains known to exist in a natural product can be found in maitotoxin and palytoxin, produced by the dinoflagellates *Gambierdiscus toxicus* and *Ostreopsis siamensis*, respectively [1]. In contrast to algae, invertebrates produce polypeptides. The target of many marine neurotoxins is the sodium channel, though the sites of interaction and, thus, the pharmacological effects, differ among compounds (Table 1). However, there are exceptions to this—for example, saxitoxin is known to also interact with voltage-gated calcium and potassium channels, while the only target identified to date for palytoxin is the Na:K ATPase [2]. Table 1 provides a comprehensive overview of the structures of marine neurotoxins, as well as their molecular target and pharmacology.

**Table 1:** Overview of major marine neurotoxins.

Backbone Structure	LD_50_^a^, ARfD ^b^	Organisms	Health Impacts ^c^	Molecular Target	Pharmacology
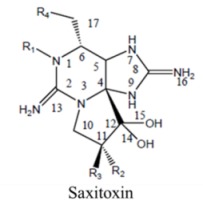					
Alkaloid	3–10, 0.7	Dinoflagellates: *Pyrodinium* *bahamense*, *Alexandrium* spp*. Gymnodinium catenatum*	PSP	Voltage-gated ion channels: Na (site 1); K; Ca	Pore blocker
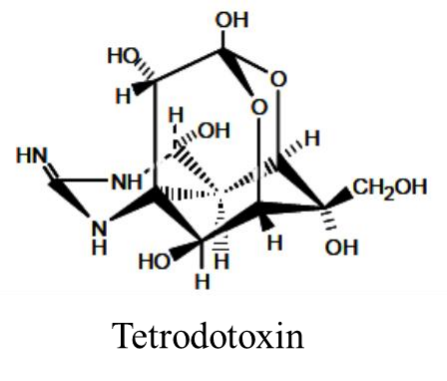					
Alkaloid	8, n/a	Fish and Bacteria	PFP	Na channel site 1	Pore blocker
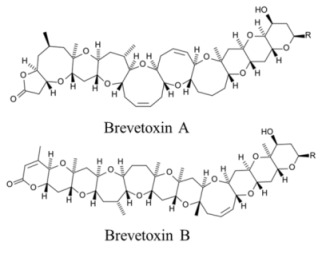					
Polyketide	170, n/a	Dinoflagellates	NSP	Na channel site 5	enhanced activation and inactivation block
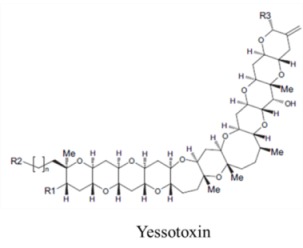					
Polyketide	380–460, 50	Dinoflagellates	NSP	Unknown	Unknown; proposed interaction with cytoskeletal
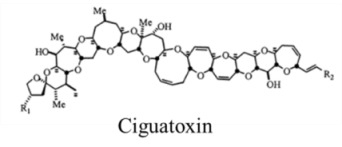					
Polyketide	0.45, n/a	Dinoflagellates	CFP	Na channel site 5	Shift in activation gating
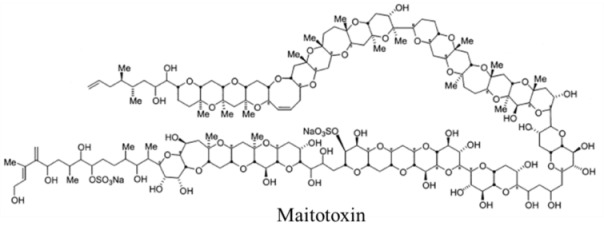					
Polyketide	0.15, n/a	Dinoflagellates	CFP	Cation channels	Channel modifier; allows non-selective ion passage
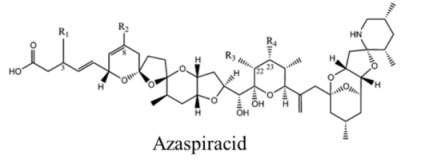					
Polyketide	200, 280	Dinoflagellates	AZP	K channel	Pore blocker
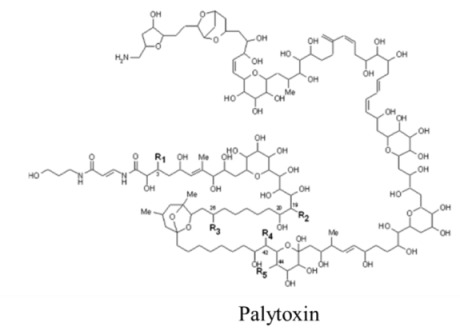					
Polyketide	0.45, n/a	Dinoflagellate	PP	Na:K ATPase	Channel modifier; allows non-selective ion passage
Cooliatoxin: structure uncharacterized					
Polyketide	1000, n/a	Dinoflagellate	Symptoms similar to NSP	Unknown	Unknown
Ostreotoxin 3: structure uncharacterized					
Polyketide	32100, n/a	Dinoflagellate	Symptoms similar to NSP	Unknown	Inactivation gating modifier
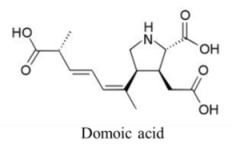					
Amino acid	3600, 100	Diatom	ASP	Glutamate receptors	Depolarization via prolonged influx of Ca^+^ and Na^+^
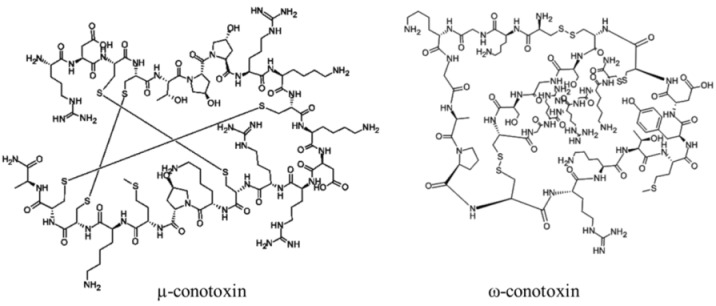					
Polypeptide	12000–30000, n/a	Cone snails	N/A	Na channel sites 1 (μ) and 6 (δ); Ca channel (ω); nicotinic acetylcholine receptors (α)	Pore blocker (μ); prolonged channel opening (δ)
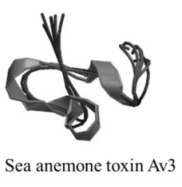					
Polypeptide	170, n/a	Sea anemones	N/A	Na channel site 3; K channel	Slow/block channel inactivation

^a^ LD_50_ (Lethal Dose 50%, the concentration of a toxic substance in a medium at which 50% of the individuals in the test die) based on mouse i.p. injection with values presented as μg/kg. ^b^ ARfD, Acute Reference Dose: the estimated amount in food that can be ingested in a 24 h period or less with no appreciable health risk to humans. ^c^ PSP, Paralytic Shellfish Poisoning; PFP, Pufferfish Poisoning; NSP, Neurotoxic Shellfish Poisoning; AZP, Azaspiracid Poisoning; ASP, Amnesic Shellfish Poisoning, CFP; Ciguatoxin Fish Poisoning; PP Palytoxin Poisoning.

Algal toxins are reported to be responsible for between 50,000 and 500,000 human intoxications per year, with an overall global mortality rate of 1.5% [1]. The toxins bioaccumulate in filter-feeding fish and shellfish, resulting in several types of human illness: paralytic shellfish poisoning (PSP), amnesic shellfish poisoning (ASP), diarrheic shellfish poisoning (DSP), ciguatera shellfish poisoning (CFP) and azaspiracid shellfish poisoning (AZP) (Table 1). In addition to the human illnesses caused by ingestion of contaminated seafood, several marine toxins possess the potential for use in bioterrorism, including the conotoxins and tetrodotoxin [3].

Among marine neurotoxins, saxitoxin is unique in that it is produced by organisms encompassing two kingdoms of life inhabiting different aquatic systems. Eukaryotic dinoflagellates are the predominant producers in marine systems [4,5,6,7], while five genera of cyanobacteria are the source in freshwater systems [8,9,10,11,12], though the route of biosynthesis is similar among the two [13]. Saxitoxin is also unique in that it is the only marine natural product classified as a Schedule I Chemical Warfare Agents per the Chemical Weapons Convention of 1993. Several excellent reviews exist on saxitoxin in freshwater systems [14,15]; thus, this review focuses primarily on the toxin as it relates to marine dinoflagellates. Here, we discuss recent advances in the field of saxitoxin research, including the identification of genes involved in its biosynthesis, potential new molecular targets and the proposed ecological role this compound may serve for the species that produce it. We also address the different means of detection, with a special emphasis on recent molecular assays designed for real-time water quality monitoring.

## 2. Saxitoxin and Derivatives: Structure and Chemistry

Saxitoxin is the parent molecule in a class of compounds, typically referred to as paralytic shellfish toxins (PSTs). Its basic structure is that of a trialkyl tetrahydropurine, with positions 2 and 8 of the purine ring containing the NH_2_ groups, which form the two permanent guanidinium moieties [16]. It possesses two pKa’s of 8.22 and 11.28, which belong to the 7,8,9 and 1,2,3 guanidinium groups, respectively [17,18,19]. At physiological pH, the 1,2,3-guanidino carries a positive charge, whereas the 7,8,9-guanidino group is partially deprotonated [18,20]. Variations in functional groups at four defined positions around the ring define the different divisions (Table 2). The divisions consist of the carbamate toxins, all of which have a carbamoyl at the R1 position, the *N*-sulfocarbamoyl toxins, the decarbamoyl toxins and the deoxydecarbamoyl toxins. Substitutions at N-1 and/or C-11 result in a decrease in toxicity relative to saxitoxin, except GTX-III, which exhibits toxicity comparable to that of saxitoxin [21]. The toxicity of the derivatives varies by approximately two orders of magnitude [22], with saxitoxin (STX) being the most toxic followed by neosaxitoxin and gonyautoxins 1 and 3. Different values have been reported in the literature; as these values are dependent in part upon the purity of the compounds, it is likely these differences are simply a result of differences in the purities of the toxin preparations [23].

**Table 2:** Molecular structure of saxitoxin and derivatives produced by marine dinoflagellates [1].

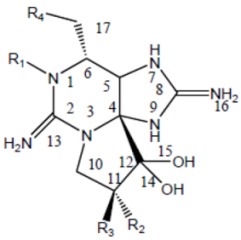					
Division	Name ^a^	R1	R2	R3	R4
	STX	H	H	H	OCONH_2_
	NeoSTX	OH	H	H	OCONH_2_
	GTX1	OH	OSO_3_^−^	H	OCONH_2_
Carbamate	GTX2	H	OSO_3_^−^	H	OCONH_2_
	GTX3	H	H	OSO_3_^−^	OCONH_2_
	GTX4	OH	H	OSO_3_^−^	OCONH_2_
	GTX5 (B1)	H	H	H	OCONHSO_3_^−^
	GTX6 (B2)	OH	H	H	OCONHSO_3_^−^
	C1	H	OSO_3_^−^	H	OCONHSO_3_^−^
*N*-sulfocarbamoyl	C2	H	H	OSO_3_^−^	OCONHSO_3_^−^
	C3	OH	OSO_3_^−^	H	OCONHSO_3_^−^
	C4	OH	H	OSO_3_^−^	OCONHSO_3_^−^
	dcSTX	H	H	H	OH
	dcNeoSTX	OH	H	H	OH
	dcGTX1	OH	OSO_3_^−^	H	OH
Decarbamoyl	dcGTX2	H	OSO_3_^−^	H	OH
	dcGTX3	H	H	OSO_3_^−^	OH
	dcGTX4	OH	H	OSO_3_^−^	OH
	doSTX	H	H	H	H
Deoxydecarbamoyl	doGTX2	H	H	OSO_3_^−^	H
	doGTX3	H	OSO_3_^−^	H	H

^a^ Abbreviations: STX, saxitoxin; GTX, gonyautoxin.

## 3. Molecular Targets and Pharmacology

### 3.1. Ion Channel Structure

The primary target of most marine neurotoxins is the sodium channel, a voltage-gated ion channel protein comprised of one principal alpha subunit (220–260 kDa) and one to three smaller (33–36 kDa) beta subunits [24]. Additionally, saxitoxin and its derivatives are also known to target the potassium and calcium channels, though the mechanism of action differs among the three. Ion channels are membrane proteins containing a pore that allows the rapid and passive diffusion of ions across the lipid bilayer. Ion channels can generally be grouped into two major classes, ligand-gated and voltage-gated, with voltage-gated channels regulated by changes in membrane potential [25]. 

**Figure 1:**
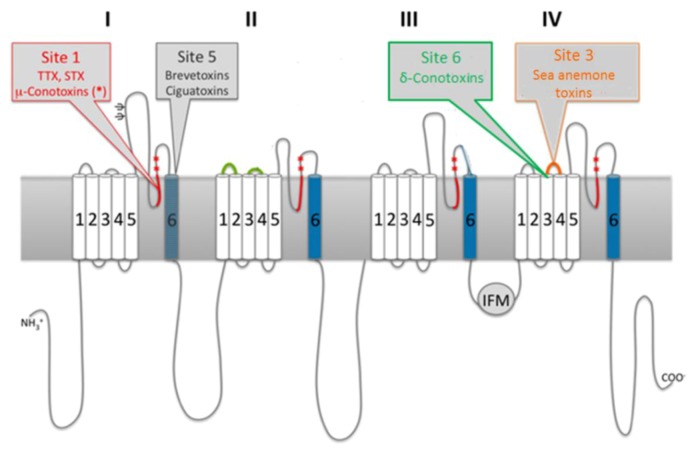
Sodium channel architecture and binding sites of marine neurotoxins. Cylinders represent transmembrane helices that comprise the four homologous domains. Sites targeted by marine neurotoxins are indicated by gray call-outs. Modified from [27].

Voltage-gated ion channels share a common structural architecture [26] (Figure 1). The alpha subunit consists of a single polypeptide with four homologous domains (I-IV). Each domain contains six transmembrane alpha helices (S1–S6) and a short reentrant segment (SS1/SS2). The S4 segment of each domain is positively charged and functions as the voltage sensor, moving outward under the influence of the electrical field and, thus, opening the pore and initiating channel activation. Each segment is connected by intra- or extra-cellular loops serving various functions. The extracellular loops between the fifth and sixth transmembrane helices of each domain, known as pore-forming (P) loops, back into the membrane to form the outer lining of the pore and the selectivity filter. The short intracellular loop connecting domains III and IV is responsible for channel inactivation [24,27]. 

### 3.2. Marine Neurotoxins Binding Sites

Pharmacological studies with neurotoxins identified six key receptor sites within sodium channels; thus, the molecular mechanisms of neurotoxins can be broadly classified into one of three groups based on their receptor site and functional effect: pore-blocking toxins, toxins that affect gating from sites within the membrane and toxins that affect gating from extracellular sites [24]. Pore-blocking toxins inhibit ion conductance via binding to the outer mouth of the pore (site 1). Toxins that alter voltage gating by binding to intramembrane receptor sites (sites 2, 5) bind to the channel when in its active state. This results in persistent activation, due to allosteric modulations that prevent an inactivation. Voltage-sensor trapping toxins alter gating via extracellular binding. These toxins bind to the S3–S4 loop of domain IV (site 3) through electrostatic interactions with specific residues. Channel inactivation is impeded, as the transmembrane segment of domain IV is held in an inward position, preventing the conformational changes needed for fast activation [28]. By targeting different receptor sites on the channels (Figure 1), marine neurotoxins exhibit different molecular pharmacologies (Table 1). 

### 3.3. Molecular Targets of Saxitoxin

#### 3.3.1. Ion Channels

The long-established molecular target of saxitoxin is the voltage-gated sodium channel in nerve and muscle cells, to which it binds with high affinity and can result in death via respiratory paralysis [29]. The toxin binds to receptor site 1, which is formed by two rings of amino acid residues located in segment SS2 of the S6 transmembrane segment in each of the four domains [30,31]—*i.e.*, the P-loops. The first ring is comprised of the residues aspartic acid (D) 384 in domain I, glutamic acid (E) 942 in domain II, lysine (K) 1423 in domain III and alanine (A) 1714, while the second ring contains E387, E945, methionine (M) 1425 and D1717 (located in domains I–IV, respectively). It has been suggested that these clusters may form ring structures that line the outer lip of the pore. 

One saxitoxin molecule binds per sodium channel [32], with the 7,8,9-guanidinium moiety as the active group involved in toxin binding. It is electrostatically attracted to the lip of the channel by fixed anionic charges [33]. Thus, saxitoxin is able to effectively block the inward flow of sodium ions into the cell, with the guanidinium able to act as a cationic substitute for the sodium ion. Therefore, saxitoxin blocks the sodium channel from the exterior of the channel and cannot exert its pharmacological action from the cell’s interior [34]. Studies of sodium channel inhibition at different pH values have shown that saxitoxin has a greater effect at neutral pH, due to protonation of its hydroxyl groups [35]. Both the guanidinium and hydroxyl groups are needed for sodium channel recognition by the toxin, as modifications near either of these moieties have resulted in loss of biological function of the toxin.

Saxitoxin also binds to the human potassium channel, though its mechanism of interaction differs from that with the sodium channel. It modifies channel gating rather than blocking the channel, resulting in stronger transmembrane depolarization for the channel to open and thus reducing overall potassium conductance [36]. Unlike its interaction with the sodium channel, in which only a single molecule binds, four or more molecules are able to bind to extracellular sites [36]. Antiquity of the saxitoxin gene cluster indicates that potassium channels, rather than sodium channels, may have been the original intended target of the compound [37]. 

Saxitoxin also acts on voltage-gated calcium channels, though the blockage is not complete as in sodium channels [38]. However, the results obtained suggest that saxitoxin acts on the calcium channel at an extracellular site, possibly an area associated with the selectivity filter [38], similar to its interaction with the sodium channel. Interestingly, voltage-gated sodium channels evolved from calcium channels and were present in the common ancestor of choanoflagellates and animals, although this channel was likely permeable to both sodium and calcium ions [39].

#### 3.3.2. Saxiphilin

Saxitoxin has also been shown to bind to saxiphilin, a *ca*. 90 kDa soluble protein isolated from bullfrog (*Rana catesbeiana*) plasma, which sequence analysis revealed is related to the transferrin family of proteins [40,41]. The majority of these proteins are iron-binding, monomeric proteins of approximately 80kDa. They possess a bilobal architecture exemplified by the high homology existing between the *N*- and *C*-terminal halves of the protein. This homology includes metal ion—typically Fe^3+^—residues located in each lobe [42]. However, saxiphilin does not contain an iron-binding site, due to a 144 residue insertion in the N lobe and many amino acid substitutions in the C lobe [41], the implications of which are discussed below. 

The saxitoxin binding site has been shown to be located within the C lobe [43]. It appears the saxitoxin binding site within saxiphilin has evolved from the transferrin Fe^3+^ binding site and may utilize some of the amino acid residues previously used to ligate the metal to now bind saxitoxin. This protein is specific for saxitoxin, as it is unaffected by tetrodotoxin and various cationic compounds [40]. As with the sodium channel, only a single saxitoxin molecule binds to saxiphilin [44], and similar thermodynamics were observed for saxitoxin binding to saxiphilin and the sodium channel [34]. Unlike sodium channel binding, protonation of the 7,8,9-guanidinium moiety is not required [44]. Saxiphilin’s isolation from frogs led to the hypothesis that it functioned as a detoxification mechanism upon toxin exposure from freshwater cyanobacteria [41].

#### 3.3.3. Copper Transporters

The copper transporter has also recently been proposed as an additional molecular target of saxitoxin. A series of studies conducted with yeast and the photosynthetic green alga, *Chlamydomonas reinhardtii*, demonstrated that exposure to saxitoxin inhibited copper uptake in both species [45]. Both possess high-affinity copper uptake systems in which the copper transporter plays a significant role [46,47,48,49]. Both share structural similarities with human Ctr, for which the projection structure reveals a design more closely resembling ion channels rather than classic transporters, including a motif on transmembrane 3 that may contribute to selectivity and gating [50]. Therefore, it has been proposed that saxitoxin may bind to copper transporters, though this remains to be experimentally verified.

In examining the structural properties of the above proteins in relation to saxitoxin binding, the theme of metal binding sites emerges. As discussed, it appears that the saxitoxin binding site within saxiphilin has evolved from an Fe^3+^ binding site, with some of the amino acid residues previously used to ligate the metal to now bind saxitoxin [34]. It has been suggested that saxitoxin may be potentially considered a substitute ligand for Fe^3+^ [44]. Pioneering studies examining saxitoxin binding in sodium and potassium channels showed that the toxins acted at a metal cation binding site, with several monovalent cations able to compete reversibly with the toxins for their binding sites [51]. The *N*-terminal domain of yeast and algal copper transporters have been well-characterized and are known to be enriched in metal-binding amino acids, such as Met, His and Cys [49,52], providing a platform from which to examine saxitoxin’s binding ability. 

## 4. Genetics of Saxitoxin Production

### 4.1. Dinoflagellate Genome Overview

Though the capacity for toxin production is scattered across both freshwater prokaryotes (cyanobacteria) and marine eukaryotic (dinoflagellate) species, the pathway for saxitoxin biosynthesis is believed to be similar between the two [13] and was initially proposed based on extensive studies using labeled precursors with the dinoflagellate *Alexandrium tamarense* and the cyanobacterium *Aphanizomenon flos-aquae* [13,53]. However, genetic information [54], coupled with screening of the biosynthetic intermediates and the *in vitro* biosynthesis of saxitoxin [55], has resulted in modifications of the original pathway. These modifications occur primarily in the initial steps of biosynthesis, though still include the rare chemical reaction involving a Claisen-type condensation on arginine. 

Saxitoxin biosynthesis genes were first identified in the toxic freshwater cyanobacteria, *Cylindrospermopsis raciborskii* T3 [54], followed by *Anabaena circinalis* (AWQC131C), *Aphanizomenon* sp. NH-5 [56] and *Lyngbya wollei* [57]. Until recently, the extremely large (*ca*. 3–245 Gb of DNA, the equivalent of 1- to 80-fold as much as a haploid human cell) and complex (highly redundant, with high gene copy number) [58] genomes of dinoflagellates have posed significant challenges in identifying toxin-related genes. The dinoflagellate genome contains the largest number of nuclear genes of all unicellular eukaryotes. These genes occur in complex families, most of which evolved via recent duplication events [59]. A genome size *versus* gene content regression study predicted over 42,000 genes in the smallest dinoflagellate genome and over 92,000 in the largest [60]. Global transcriptome studies revealed that toxic *Alexandrium* spp. contain *ca*. 40,000 transcribed genes, making it the most complex protist transcriptome to date, with many transcripts resulting from sequence variants of individual genes, further contributing to the transcriptional complexity of the dinoflagellate genome [59,61].

### 4.2. Dinoflagellate Saxitoxin Biosynthesis Genes

**Figure 2:**
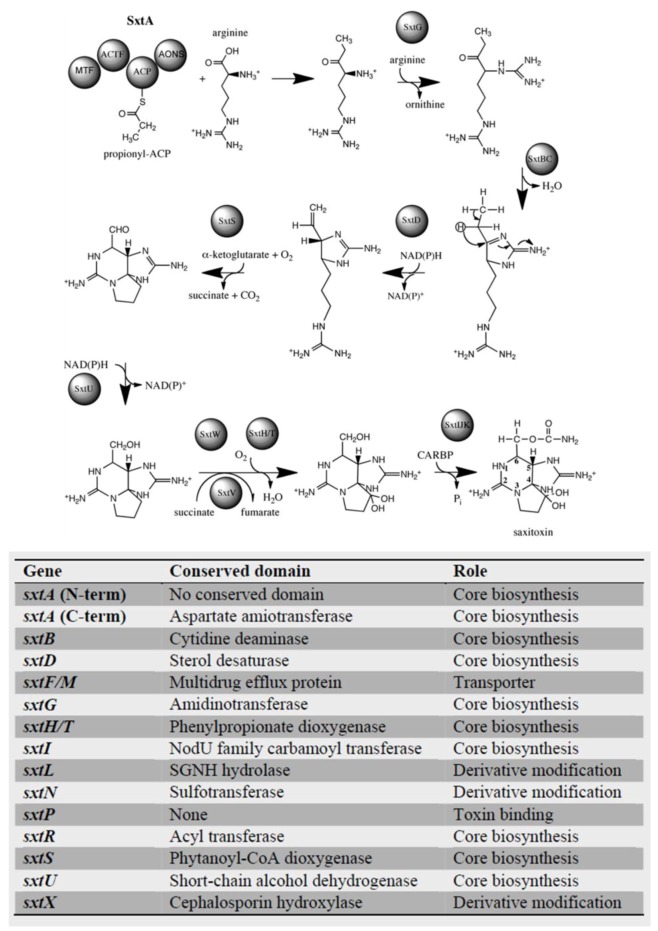
Saxitoxin genes identified in dinoflagellates. Saxitoxin biosynthesis pathway and genes involved based on studies in cyanobacteria [37]. Dinoflagellate genes identified to date are listed below the figure and are cumulative results of high-throughput sequencing experiments.

Through the use of high-throughput sequencing technologies, saxitoxin biosynthesis genes have recently been identified in multiple species of dinoflagellates (Figure 2) [62,63], including representatives from all three (*Alexandrium*, *Pyrodinium*, *Gymnodinium*) toxin-producing genera. All genes directly implied in toxin synthesis in cyanobacteria have also been identified in dinoflagellates, along with genes related to toxin transport and modification [63] (Figure 2). The findings with *C. raciborskii* T3 revealed that saxitoxin biosynthesis is initiated by SxtA, a novel polyketide synthase [54]. SxtA performs the following steps: the loading of the acyl carrier protein (ACP) domain with acetate from acetyl-CoA and methylation of acetyl-ACP to propionyl-ACP, followed by the aminotransferase domain of SxtA, then performing a Claisen condensation of propionyl-ACP with arginine. Two different types of transcripts have been recovered for dinoflagellate *sxtA*, with differences occurring in sequence, length and number of domains [62]. One set contained only three *sxtA* domains, while the second contained the four typically encoded by cyanobacterial *sxtA*. Transcripts contained features typically associated with eukaryotic genes, notably the presence of poly(A)-tails at the 3′-end and spliced-leader sequences at the 5′-end, demonstrating that *sxtA* genes are encoded in the dinoflagellate nucleus, and thus, toxin synthesis does not originate from co-cultured bacteria. One hundred to two hundred forty copies of the *sxtA4* domain exist in the genome [62], in keeping with the general feature of dinoflagellate genes occurring in multiple copies [64,65]. Unlike the cyanobacterial *sxtA*, which was shown to result from the fusion of proteo- and action-bacterium proteins [66], the two domains are not fused in dinoflagellates and, in fact, are encoded by different proteins [63]. 

Sequences with similarities to the *N*-terminal portion of *sxtA*, containing the acyltransferase and phosphopantetheinyl-attachment site (PP-binding) domain, as in cyanobacteria, have been found in the saxitoxin-producing species *A. tamarense* Groups I and IV, *A. catenella* and *P. bahamense*, as well as in the non-toxin producing *A. tamarense* Group III, indicating their functions may not be limited to saxitoxin production [63]. Additionally, homologs of the *C*-terminus aminotransferase domain of *sxtA* and *sxtG* were found exclusively in toxic species, including *P. bahamense* and *G. catenatum*, indicating they may be unique to toxic species. Overall, transcriptome analyses from multiple toxin-producing dinoflagellate spp. suggest that the genes involved in the first three steps of saxitoxin biosynthesis (*sxtA*, *sxtG*, *sxtB*) in dinoflagellates and cyanobacteria are derived from common ancestral proteins not involved in saxitoxin synthesis, as they are found in organisms that do not produce the toxin [63]. When considering the complexities of dinoflagellate gene organization and regulation, it is understandable that while the core toxin biosynthesis genes have been identified, associated genes may not possess similar functional or transcriptional properties to known cyanobacterial genes. It is also likely that, in dinoflagellates, genes associated with toxin biosynthesis serve other functions as well, as phylogenomic analysis reveals that many toxin-related genes are widely distributed among dinoflagellates [67].

### 4.3. Saxitoxin-Related Enzymes

In addition to the recent identification of core toxin synthesis genes from multiple species of dinoflagellates, two enzymes displaying activities specific to saxitoxin and its derivatives have also been purified and characterized from toxic *G. catenatum*. Yoshida *et al.* [68] purified a sulfotransferase, which transferred a sulfate group to O-22 of hydroxy derivatives (11-α,β-hydroxy saxitoxin), while a sulfotransferase purified by Sako *et al.* was specific to N-21 of saxitoxin and gonyautoxin 2 + 3 and did not exhibit O-22 sulfation [69]. Of the three toxic genera, comprehensive transcriptomic analyses have been performed for *Alexandrium* spp., with lower coverage transcriptomes obtained for *P. bahamense* and *G. catenatum*. Thus, it is not surprising that genes coding for these enzymes have not yet been identified.

## 5. Effects of Saxitoxin on Human and Environmental Health

Saxitoxin and its congeners are the causative agents of paralytic shellfish poisoning (PSP) and, as determined recently, saxitoxin pufferfish poisoning (SPFP) [70]. In the case of PSP, filter-feeding mollusks (typically bivalves) and crustaceans ingest the toxic cells, concentrating the toxins within the organs and tissues. Although mussels and clams are the dominant vectors for PSTs, there are increasing reports of non-traditional organisms, such as gastropods, crustaceans and certain fish, also serving as vectors [71]. The first reported PSP event occurred in 1927 near San Francisco, USA, and was caused by *A. catenella*, resulting in 106 human illnesses and six deaths [1]. Since that time, members of the *Alexandrium*, *Gymnodinium* and *Pyrodinium* genera have all been reported as major sources of PSTs. While most PSP outbreaks result from the consumption of contaminated shellfish, the degree of intoxication varies. Toxicity levels fluctuate among bivalve species, due to differences in the toxin components retained and the rate of depuration, as some species depurate toxins rapidly, whereas others are slow to detoxify [72]. Symptoms of PSP include paresthesia and numbness, first around the lips and mouth and then the face and neck, muscular weakness, sensation of lightness and floating, ataxia, motor incoordination, drowsiness, incoherence and progressively decreasing ventilator efficiency. In cases of severe intoxication, PSP leads to respiratory paralysis and death [72]. On a global basis, almost 2000 cases of human PSP are reported per year, with a 15% mortality rate [73]. The geographical distribution of these cases is related to the global distribution of the various PST-producing species and their toxigenic strains [74]. While numerous fatal cases of PSP have been reported globally, the successful implementation of monitoring programs in many countries has helped to minimize health risks and reduce human illnesses and fatalities [71]. 

If PSTs ingested by fish or other secondary producers are not lethal to those organisms, the possibility exists for bioaccumulation and passage up the food chain. Through this process, PSTs have also been confirmed or implicated in the deaths of sea birds, whales and monk seals [74]. In the cases of mass mortality events involving birds, it is mostly piscivorous birds that are affected after consuming fish contaminated by PSTs. In May 1942, at least 2000 dead birds were observed along the coastal beaches of Washington State, USA. This coincided with an *A. catenella* bloom that resulted in six human cases of fatal PSP. PSTs have also been implicated or confirmed as the source of mortality in humpback whales and monk seals. During a five-week period beginning in 1987, 14 humpback whales (*Megaptera novaeangliae*) died off the waters of New England, USA, after eating Atlantic mackerel (*Scomber scombrus*) containing PSTs. More recently, from May to July 1997, at least 117 Mediterranean monk seals (*Monachus monachus*) died along the coast of the western Sahara, Africa. Water samples indicated the presence of the known PST producers, *A. minutum* and *G. catenatum*, and variable levels of PSTs were found in all seal samples tested [74].

Saxitoxin PFP is a similar illness, expect that bioaccumulation occurs in puffer fish rather than shellfish. From January 2002 to May 2004, 28 puffer fish poisoning (PFP) cases in Florida, New Jersey, Virginia and New York were linked to puffer fish (*Sphoeroides* spp.) harvested from the Indian River Lagoon (IRL), Florida, USA. Saxitoxin and two of its derivatives were determined to be the active toxins, with *P. bahamense* identified as the source. This lead to the characterization of the food poisoning syndrome as saxitoxin puffer fish poisoning (SPFP) to distinguish it from PFP, which is traditionally associated with tetrodotoxin and from PSP [70]. These findings led to a permanent ban on puffer fish harvesting along the east coast of Florida along with the establishment of a monitoring program to determine the distribution and concentrations of PSTs in various puffer fish species. This monitoring program found that STX concentrations from fish tissue averaged greater than 20-times the action limit for shellfish, with maximum values exceeding 200-times the action limit [75]. 

## 6. Methods of Detection

Methods for toxin analysis can be grouped into five broad headings: *in vivo* animal bioassays, analytical techniques, *in vitro* functional assays, *in vitro* cell assays and immunoassays (ELISA). In addition to methods for toxin detection, molecular tools targeting toxin-producing species are also becoming increasingly common and are therefore included here. 

### 6.1. *In Vivo* Animal Bioassays

The current “gold standard” for saxitoxin measurement is the mouse bioassay (MBA). The MBA has been refined and standardized by the Association of Official Analytical Chemists to provide quick and adequately accurate measurements. However, this method is becoming increasingly criticized, due to its use of live animals, and alternatives have recently been accepted or are undergoing validation testing. The potency, in some literature, referred to as relative toxicity, of saxitoxin is typically measured via the mouse bioassay. This assay entails injection (typically intraperitoneal) of a 1 mL test solution into a mouse weighing 17–23 g and observing the time from injection to death. The number of mouse units is obtained by reference to a standard table that incorporates the death time and mouse weight, with one mouse unit being defined as the amount of toxin that will kill a 20 g mouse in 15 min [22]. This corresponds to 200 ng of saxitoxin [76]. Interferences include samples with high salt content, which suppresses toxic effects [77], and zinc accumulation in oysters, which can results in mouse death at concentrations not detrimental to human health [78]. Further investigations into the effects of metals on the MBA revealed that they exhibited a suppressive effect [79]. Additionally, factors, such as gender and mouse strain, have been shown to produce significant differences of up to seven-fold in neurotoxins [80]. In most countries, the action level at which fisheries are closed is 80 μg STX equivalents/100 g shellfish [81] (this value is also frequently expressed in the literature as 400 MU/100 g), though this value has been lowered to 30 and 40 μg STX eq/100 g shellfish in Mexico and the Philippines, respectively [82]. The United States Food and Drug Administration alert level for saxitoxin is 80 μg/100 g shellfish meat, and so, commercial shellfish harvesting in the US must be suspended if higher concentrations are detected in routine monitoring programs [83]. The limit of detection of the MBA is *ca*. 40 μg STX eq/100 g shellfish. A variation of this assay has recently been proposed that utilizes sublethal indicators of toxicity rather than time to death as a means of screening for the toxins. It is based on levels of the neurotransmitter, acetylcholine, in the blood of the mice at timed intervals [84]. The detection limit of the assay was less than 1 μg/kg weight (equivalent to 20 ng/mL), which is below the regulatory limit required for shellfish closures. 

### 6.2. Analytical Techniques

Analytical methods can be used to both detect and quantify the toxins. High performance liquid chromatography (HPLC), widely used for the separation of organic compounds, was one of the first analytical methods developed for saxitoxin detection and is now routinely used. The basis of the HPLC method for saxitoxin analysis was established in the late 1970s using a post-column derivatization with a silica-based stationary phase. Since that time, many methods for toxin separation have been developed using both pre- and post-column oxidation with a variety of columns. One of the initial challenges in toxin detection was the lack of any useful UV absorption, which was alleviated by the conversion of the toxins to fluorescent derivatives [85]. Since the 1970s, a variety of modifications have led to improved separation and detection of the different congeners, including sample extraction, type of column, eluent composition and oxidation processes [82]. Based on a collaborative study encompassing 16 labs from 12 countries, it was recommended that the method for quantitative determination of PSTs in shellfish using pre-chromatographic oxidation should be accepted by the AOAC International as an official method, and it was accepted in the same year [82]. The recent advances in HPLC methods have been made and approved for use by monitoring agencies, such that the official alternative technique to the MBA in the European Union is a LC–fluorescence detection (LC-FLD) method (AOAC 2005.06) [86]. 

Liquid chromatography-mass spectrometry (LC-MS) is a powerful technique that allows the identification on unknown compounds, quantification of known materials and elucidation of structural properties of molecules. It is becoming used more routinely for not only PSTs, but all marine toxins analyses, to the point that it has been proposed as the universal method for all marine toxins [82]. An additional method that is used in conjunction with mass spectrometry is hydrophilic interaction liquid chromatography-tandem mass spectrometry (HILIC-MS/MS). First developed in the Quilliam lab for both PSTs and other algal toxins [87], a single run was able to separate and detect all major PSTs in ranges between 50 and 1000 and 5–30 nM, depending on type of system [88]. Further developments have enabled detection limits in concentration ranges of 5–50 nM and 25–200 nM, with excellent linearity [89].

### 6.3. *In Vitro* Functional Assays

The receptor binding assay for toxin detection presents an alternative to the MBA for routine monitoring. First introduced in 1984 [90] and further developed and adapted to microtiter plate format over the years [91,92], this assay is based on the interaction between the toxins and one of their pharmacological targets, site 1 of the sodium channel. In the assay, tritiated saxitoxin competes with unlabeled saxitoxin (and/or its derivatives) for a finite number of receptor sites on the voltage-gated sodium channel in a rat membrane preparation. Upon establishment of binding equilibrium, unbound labeled saxitoxin is removed and the receptor-bound labeled saxitoxin quantified by liquid scintillation counting, with the reduction of labeled saxitoxin proportional to the amount of unlabeled toxin present. Using the microtiter plate format, a detection limit of 5 ng STX eq/mL was achieved, with a strong predictive value for toxicity, as per the MBA [92]. With the availability of standards, Usup *et al.* [93] explored the feasibility of this assay for detection and quantification of the various congeners based on competitive binding curves. Receptor binding affinity occurred in the order STX > GTX1/4 > neoSTX > GTX2/3 > dcSTX > GTX5, similar to the order of toxicity obtained with the mouse bioassay. Most recently, an international, multi-lab study determined that the Receptor Binding Assay was able to accurately detect PSTs in the critical range of shellfish toxicities below, near and slightly above the regulatory limit in comparison with the MBA, demonstrating its suitability for routine monitoring of shellfish [94]. 

### 6.4. *In Vitro* Cell Assays

Several cell viability assays were developed concurrently that utilized the antagonist effects of the channel-blocking PSTs in combination with veratridine and ouabine. These assays were modified from that developed by Kogure *et al.* [95], which was based on the ability of tetrodotoxin (TTX) to protect mouse neuroblastoma cells treated with veratridine and ouabine. Veratridine is a sodium channel activator that causes an influx of sodium ions, leading to cell swelling and eventual lysis, while ouabine blocks the action of Na/K-ATPases. TTX prevents the influx of sodium ions and protects the cell from swelling and eventual death. The basis of Kogure’s assay was microscopy: counting the number of rounded cells after toxin treatment. However, this assay was criticized, due to the varied morphology of the mouse neuroblastoma cells and, thus, the inherent difficulty in counting which cells were rounded based on sodium uptake. The assays developed by Jellet *et al.* [96], Gallacher and Birkbeck [97] and Manger *et al.* [98] employed colorimetric endpoints based on direct cell staining, vital stain uptake and tetrazolium dye reduction, respectively, allowing for automation and use in 96-well plate format. With these assays, a detection limit of 10 ng STX eq/mL extract (2.0 μg STX eq/100 g shellfish tissue) was achieved [96]. The commercially-available MIST (Maritime *In vitro* Shellfish Test) [99] was developed from this neuroblastoma cell bioassay [96] and comes in multiple formats suitable for quantitative or qualitative screening.

Another type of *in vitro* cell assay has been developed that is based on the detection of toxin-induced membrane depolarization through the use of voltage-sensitive fluorescent dyes. Nicholson *et al.* [100] developed a membrane potential assay that monitored the output of the voltage-sensitive fluorescent probe rhodamine 6D in mouse synaptoneurosomal fractions. The assay measures the ability of saxitoxin to inhibit veratridine-increased rhodamine 6G fluorescence as a means of quantifying the toxin block of depolarization as a result of sodium channel opening. The assay developed by Louzao *et al.* [101] is also based on the antagonistic effects of veratridine and saxitoxin on membrane potential, with changes in membrane potential detected with the fluorescent probe bis-oxonol. The authors reported that this assay, performed in microplate format, was able to detect saxitoxin and derivatives at <1 ng STX eq/mL. Using the fluorescent probe bis-(1,3-diethylthiobarbituric acid)trimethine oxonol (DiSBAC_2_(3)), a method using flow cytometry was developed, enabling detection within minutes down to 1 ng STX eq/mL, with a nearly linear dose response curve between 1 and 100 ng STX eq/mL [102]. While these functional assays using fluorescent probes provide promise for sensitive and rapid detection of saxitoxin and its derivatives, further validation is required for acceptance of these techniques in the future [82]. 

### 6.5. Immunoassays

Enzyme-linked immunosorbent assays (ELISAs) are a type of biochemical assay that utilize antibodies raised to the analyte of interest, with detection typically manifested as a color change. A range of assays have been developed for saxitoxin and several of its derivatives that utilize both direct and indirect formats, with poly- and mono-clonal antibodies. Using polyclonal antibodies, Usleber *et al.* [103] developed both microtiter and test strip assays for saxitoxin. Detection limits of 7 pg/mL for the microtiter-based ELISA and 200 pg/mL in the test strip using visual evaluation were achieved, with detection limits of 3 and 4 ng/g shellfish tissue, respectively [103]. Chu *et al.* developed an indirect ELISA with antibodies for neoSTX [104] and found poor correlation between assays based on either anti-STX antibodies or anti-neoSTX antibodies. However, combining the two assays improved the detection rate to the extent that the assays could be used to screen out 80%–85% of MBAs that produced negative or low positive results. Since then, additional indirect assays have been developed for STX and neoSTX, with a detection limit of 10 pg/mL [105,106]. Direct assays have also been developed for STX, neoSTX and GTX2/3, with detection limits in the pg/mL range [106,107,108,109].

As the ELISA platform has been well-developed over the years, several commercial kits are now available. These include the Ridascreen fast saxitoxin test (R-BioPharm), the Abraxis ELISA for PSP (Abraxis) and the MaxSignal Saxitoxin ELISA (Bio Scientific). The Ridascreen assay is tailored specifically for shellfish testing, having a much higher detection limit than the Abraxis and MaxSignal assays (50 *versus* 0.02 μg and 1.2 μg/L, respectively). One of the concerns with ELISAs is cross-reactivity within the different derivatives, which is not a hindrance if cross-reactivity correlates with toxicity. For example, the C-toxins have low cross-reactivity, but also low toxicity, and so, the assay outcome can indicate actual toxicity. This is not the case with the highly toxic decarbamoyl and N1-hydroxylated variants, which the assays do not detect. Due to these concerns, it is recommended that ELISAs be used as screening tools rather than quantitative assays [82]. 

### 6.6. Molecular Tools

With the recent identification of genes associated with toxin production comes the development of molecular tools for detecting the presence of toxin-producing species. Several quantitative polymerase chain reaction (PCR) assays have been developed targeting ribosomal RNA gene regions of known toxin-producing species. These include an assay based on molecular beacon chemistry targeting the hypervariable region of the large subunit (28S) rRNA gene for *A. catenella* [110]. A quantitative PCR assay utilizing SYBR Green chemistry was developed that targets the aminotransferase domain (*sxtA4*) of the core *sxtA* gene in both *Alexandrium* and *Gymnodinium* spp. and is applicable to environmental samples, demonstrating a positive correlation with microscopic observations [111]. Most recently, a quadruplex qPCR assay was developed for detecting several cyanobacterial toxin biosynthesis genes, of which saxitoxin was included [112]. 

An assay targeting the D1/D2 region of the 28S rRNA gene of toxic *Alexandrium* spp. was developed that utilizes the LAMP (loop-mediated isothermal amplification) method [113]. This technique, in which DNA is rapidly amplified under isothermal conditions [114,115,116], consists of incubating a mixture of template DNA, six different primers, DNA polymerase with strand displacement activity and substrates at a constant temperature between 60 °C and 65 °C. The target gene is detected by the increase in the turbidity of the reaction mixture, which coincides with the production of precipitate correlated with the amount of amplicon. The assay was capable of detecting single cells of toxic *Alexandrium* spp. from environmental samples. 

Whole cell fluorescent *in situ* hybridization (FISH) probes based on the ribosomal RNA sequences have been developed to distinguish between toxin- and non-toxin-producing *Alexandrium minutum* strains [117]. Additionally, DNA probes targeting the 28S rRNA of toxin-producing *Gymnodinium catenatum* have been developed for use in FISH or sandwich hybridization assays [118].

## 7. Ecological Functions

Due to their detrimental effects on human health, saxitoxin and its derivatives are often described as potent neurotoxins. However, it is highly unlikely that the original intended target(s) of the toxins were mammalian ion channels. Dinoflagellates are an ancient group: the first evidence of their existence dates back to the early Triassic period (245–208 million years ago). The *sxt* gene cluster likely emerged at least 2100 Mya [37], at a time when organisms had not yet evolved voltage-gated sodium channels [37]. Phylogenetic analyses of key saxitoxin-related genes suggest that the saxitoxin biosynthesis pathway was assembled independently in dinoflagellates and cyanobacteria, albeit using some evolutionarily-related proteins [63], providing further support as to an eco-evolutionary role unrelated to the current molecular targets of the toxin. Thus, toxin production likely did not emerge for the purpose of the blocking of the sodium channel in humans and other mammals. It is quite feasible that these molecules are not intended to serve a toxic role or, as is the case with tetrodotoxin, that the toxin even has the same function among different species [119]. Tetrodotoxin, similar to saxitoxin in both structure and function, is widely distributed among the animal kingdom, with varied functions: in the blue ringed octopus (*Hapalochlaena maculosa*) and tropical flatworms, it is used to paralyze the prey; while in pufferfish (*Fugu niphobles*) it functions as a pheromone [119,120]. 

A multitude of hypotheses have been proposed as to the role these toxins serve. PSTs have been suggested to function as pheromones in *Alexandrium* population dynamics, potentially regulating mating and the induction of cyst formation [119]. They share several key characteristics with demonstrated pheromones that implores to the logic of this hypothesis [121], including low levels of secretion (10^−9^–10^−1^^0^ M) and profiles similar to that of terrestrial animal species, in which a mixture of compounds, rather than a single species, is produced. As very little (<5%) of the toxin is released from the cells during exponential growth, these water-soluble, but stable, compounds must be released during senescence, which would coincide with the induction of sexuality during bloom decline [121]. This hypothesis accounts for the wide diversity in toxin profiles among geographical populations and strains, as well as the high stability of the toxin profile within a strain, which is necessary to ensure fidelity within and among mating groups. What is not explained by this hypothesis, however, is how mating responses are mediated in nontoxigenic strains of the same species [121]. Immunolocalization of saxitoxin in the nucleus of toxic dinoflagellates in close proximity to the chromosomes [122] suggests that it may play a role in chromosome structural organization; the two positively-charged guanidinium groups may bind in a manner analogous to that of polyamines or other divalent cations. However, the question then becomes: what molecule functions as the substitute for saxitoxin in non-toxic cells? Due to the large number of nitrogen atoms within saxitoxin, it has also been speculated that the molecule may play a role in nitrogen storage, though this suggestion has been countered with the fact that nitrogen storage would be accomplished in a more bioenergetically efficient fashion through the use of lower molecular weight compounds, such as amino acids or urea [123].

The most popular and well-studied hypothesis as to the role of saxitoxin is that it functions as a grazing deterrent. Many different organisms, including multiple zooplankton species, larval and adult fish and larval and adult macroinvertebrates have all been shown to feed on PST-producing dinoflagellates; however, its effects have shown a range of results, some of which support and others which refute the hypothesis [124]. Numerous studies on the bioaccumulation of saxitoxin have shown the ability of bivalves and crustaceans to accumulate high levels of the toxin [9,125,126], in effect refuting its role as a grazing deterrent in mollusks. Support for a grazer-deterring function of PSTs was provided by Selander *et al.* [127], who demonstrated that PST-producing *A. minutum* increased their cellular toxin content in response to waterborne cues from zooplankton grazers, resulting in an increased resistance to copepod grazing. An extension of this work demonstrated that grazer-induced PST production in *A. minutum* also possessed a high degree of specificity to cues from the different grazers, leading to the suggestion that variations among the history of coexistence between *A. minutum* and the different grazer species could be involved [128]. Contradicting results exist in the literature as to whether zooplankton species actively reject toxic cells. Some results suggest that dinoflagellate cells containing PSTs could be discerned by copepods prior to ingestion [129]. However, even in studies in which multiple species of grazers exhibited little to no change in feeding behavior when presented with toxic cells, subsequent effects then included an increase in mortality and a decrease in reproductive success [130,131,132]. Overall, while the role of PSTs as a grazer deterrent has often been suggested, the specific interaction is dependent on both the toxin composition of the prey and on the grazer tested [133], and thus, this hypothesis has remained controversial [121]. One scenario stemming from the cumulative results of these studies has been that grazing pressure is alleviated in toxin-producing species allowing for bloom formation; however, recent lab-based studies refute this scenario [132]. Ecosystem modeling that included multiple species of phyto- and zoo-plankton, along with bacteria, in an enclosed system indicated that the overall impact of toxin production was small and would not cause appreciable modifications in the long-term evolution of the system [134]. Most studies presume that it is the production of saxitoxin that alters the feeding behaviors or reproductive mechanisms of zooplankton. However, both toxic and non-toxic strains of *Alexandrium* spp. are able to produce an extracellular compound with allelopathic effects on other species of dinoflagellates, the ultimate outcomes of which were loss of motility and cell lysis [135]. Similar results have been obtained with gastropod larvae: exposure to toxic or non-toxic *Alexandrium* spp. resulted in feeding inhibition and ultimate death by an as-yet unidentified compound [136]. Whatever the hypothesis, studies that seek to gain an understanding of toxin production as a response to environmental triggers and chemically-mediated species interactions will help define key chemical and molecular processes that help maintain biodiversity and ecosystem functionality [137].

## 8. Conclusions

Among marine neurotoxins, saxitoxin is unique in that it is produced by organisms encompassing two kingdoms of life inhabiting different aquatic systems: freshwater cyanobacteria and marine dinoflagellates. Until recently, the molecular biology of toxin biosynthesis in dinoflagellates remained elusive, due mainly to the complexity of their genomes. However, high-throughput sequencing technologies have enabled the recent detection of saxitoxin biosynthesis genes in all three toxin-producing genera of dinoflagellates [62,63]. Continued advancements with these technologies are likely to provide the identification of additional toxin-related genes and improved understanding of transcriptional machinery involved in dinoflagellate toxin synthesis. Phylogenetic analyses of toxin-related genes will provide additional insights into the evolution of toxin synthesis, while the identification of additional molecular targets allows for the creation of new methods for detecting and tracking of PSTs.

## References

[1] Wang D.-Z. (2008). Neurotoxins from marine dinoflagallates: A brief review. Mar. Drugs.

[2] Rein K.S., Borrone J. (1999). Polyketides from dinoflagellates: Origins, pharmacology and biosynthesis. Comp. Biochem. Physiol. B.

[3] Anderson P.D. (2012). Bioterrorism: Toxins as weapons. J. Pharm. Pract..

[4] Harada T., Oshima Y., Yasumoto T. (1982). Studies on paralytic shellfish poisoning in tropical waters: 4. Structures of 2 paralytic shellfish toxins, gonyautoxin-V and gonyautoxin-VI isolated from a tropical dinoflagellate, *Pyrodinium bahamense* var. *compressa*. Agric. Biol. Chem..

[5] Oshima Y., Hasegawa M., Yasumoto T., Hallegraeff G., Blackburn S. (1987). Dinoflagellate *Gymnodinium catenatum* as the source of paralytic shellfish toxins in Tasmanian shellfish. Toxicon.

[6] Hallegraeff G.M., Steffensen D.A., Wetherbee R. (1988). Three estuarine Australian dinoflagellates that can produce paralytic shellfish toxins. J. Plankton Res..

[7] Anderson D.M., Kulis D.M., Sullivan J.J., Hall S., Lee C. (1990). Dynamics and physiology of saxitoxin production by the dinoflagellates *Alexandrium* spp. Mar. Biol..

[8] Carmichael W.W., Evans W.R., Yin Q.Q., Bell P., Moczydlowski E. (1997). Evidence for paralytic shellfish poisons in the freshwater cyanobacterium *Lyngbya wollei* (Farlow ex Gomont) comb. nov. Appl. Environ. Microbiol..

[9] Negri A.P., Jones G.J. (1995). Bioaccumulation of paralytic shellfish poisoning (PSP) toxins from the cyanobacterium *Anabaena circinalis* by the freshwater mussel *Alathyria condola*. Toxicon.

[10] Lagos N., Onodera H., Zagatto P.A., Andrinolo D., Azevedo S.M., Oshima Y. (1999). The first evidence of paralytic shellfish toxins in the freshwater cyanobacterium *Cylindrospermopsis raciborskii*, isolated from Brazil. Toxicon.

[11] Pomati F., Sacchi S., Rossetti C., Giovannardi S., Onodera H., Oshima Y., Neilan B.A. (2000). The freshwater cyanobacterium *Planktothrix* sp. FP1: Molecular identification and detection of paralytic shellfish poisoning toxins. J. Phycol..

[12] Ferreira F.M.B., Soler J.M.F., Fidalgo M.L., Fernandez-Vila P. (2001). PSP toxins from *Aphanizomenon flos-aquae* (cyanobacteria) collected in the Crestuma-Lever reservoir (Douro river, northern Portugal). Toxicon.

[13] Shimizu Y. (1993). Microalgal metabolites. Chem. Rev..

[14] Pearson L., Mihali T., Moffitt M., Kellmann R., Neilan B. (2010). On the chemistry, toxicology and genetics of the cyanobacterial toxins, microcystin, nodularin, saxitoxin and cylindrospermopsin. Mar. Drugs.

[15] Araoz R., Molgo J., Tandeau de Marsac N. (2010). Neurotoxic cyanobacterial toxins. Toxicon.

[16] Schantz E.J., Ghazarossian V.E., Schnoes H.K., Strong F.M., Springer J.P., Pezzanite J.O., Clardy J. (1975). Structure of saxitoxin. J. Am. Chem. Soc..

[17] Rogers R.S., Rapoport H. (1980). The pK_a_s of saxitoxin. J. Am. Chem. Soc..

[18] Shimizu Y., Hsu C.P., Genenah A. (1981). Structure of saxitoxin in solutions and stereochemistry of dihydrosaxitoxins. J. Am. Chem. Soc..

[19] Schantz E.J., Lynch J.M., Vayvada G., Matsumot K., Rapoport H. (1966). Purification and characterization of poison produced by *Gonyaulax catenella* in axenic culture. Biochemistry.

[20] Strichartz G. (1984). Structural determinants of the affinity of saxitoxin for neuronal sodium channels—Electrophysiological studies on frog peripheral nerve. J. Gen. Physiol..

[21] Genenah A.A., Shimizu Y. (1981). Specific toxicity of paralytic shellfish poisons. J. Agric. Food Chem..

[22] Hall S., Strichartz G., Moczydlowski E., Ravindran A., Reichardt P.B., Hall S., Strichartz G. (1990). The Saxitoxins: Sources, Chemistry, and Pharmacology. Marine Toxins: Origin, Structure, and Molecular Pharmacology.

[23] Llewellyn L.E. (2007). Predicitive toxinology: An initial foray using calculated molecular descriptors to decribe toxicity using saxitoxin as a model. Toxicon.

[24] Cestele S., Catterall W.A. (2000). Molecular mechanisms of neurotoxin action on voltage-gated sodium channels. Biochimie.

[25] Strong M., Chandy K.G., Gutman G.A. (1993). Molecular evolution of voltage-sensitive ion channel genes: On the origins of electrical excitability. Mol. Biol. Evol..

[26] Charalambous K., Wallace B.A. (2011). NaChBac: The long lost sodium channel ancestor. Biochemistry.

[27] Stevens M., Peigneur S., Tytgat J. (2011). Neurotoxins and their binding areas on voltage-gated sodium channels. Front. Pharmacol..

[28] Catterall W.A., Cestele S., Yarov-Yarovoy V., Yu F.H., Konoki K., Scheuer T. (2007). Voltage-gated ion channels and gating modifier toxins. Toxicon.

[29] Catterall W.A., Anderson D.M., White A.W., Baden D.G. (1985). The Voltage Sensitive Sodium Channel: A Receptor for Multiple Neurotoxins. Toxic Dinoflagellates.

[30] Noda M., Suzuki H., Numa S., Stuhmer W. (1989). A single point mutation confers tetrodotoxin and saxitoxin insensitivity on the sodium channel II. FEBS Lett..

[31] Terlau H., Heinemann S.H., Stuhmer W., Pusch M., Conti F., Imoto K., Numa S. (1991). Mapping the site of block by tetrodotoxin and saxitoxin of sodium channel II. FEBS Lett..

[32] Hartshorne R.P., Catterall W.A. (1984). The sodium channel from rat brain—Purification and subunit composition. J. Biol. Chem..

[33] Kao C.Y., Walker S.E. (1982). Active groups of saxitoxin and tetrodoxin as deduced from actions of saxitoxin analogs on frog muscle and squid axon. J. Physiol..

[34] Llewellyn L.E. (2006). Saxitoxin, a toxic marine natural product that targets a multitude of receptors. Nat. Prod. Rep..

[35] Baden D.G., Trainer V.L., Falconer I. (1993). The Mode and Action of Toxins and Seafood Poisoning. Algal Toxins in Seafood and Drinking Water.

[36] Wang J.X., Salata J.J., Bennett P.B. (2003). Saxitoxin is a gating modifier of hERG K+ channels. J. Gen. Physiol..

[37] Murray S.A., Mihali T.K., Neilan B.A. (2011). Extraordinary conservation, gene loss, and positive selection in the evolution of an ancient neurotoxin. Mol. Biol. Evol..

[38] Su Z., Sheets M., Ishida H., Li F.H., Barry W.H. (2004). Saxitoxin blocks L-type *I*_Ca_. J. Pharmacol. Exp. Ther..

[39] Zakon H.H. (2012). Adaptive evolution of voltage-gated sodium channels: The first 800 million years. Proc. Natl. Acad. Sci. USA.

[40] Mahar J., Lukacs G.L., Li Y., Hall S., Moczydlowski E. (1991). Pharmacologiocal and biochemical properties of saxiphilin, a soluble saxitoxin-binding protein from the bullfrog (*Rana catesbeiana*). Toxicon.

[41] Morabito M.A., Moczydlowski E. (1994). Molecular cloning of bullfrog saxiphilin—A unique relative of the transferrin family that binds saxitoxin. Proc. Natl. Acad. Sci. USA.

[42] Gaffney J.P., Valentine A.M. (2012). Beyond bilobal: Transferrin homologs having unusual domain architectures. Biochim. Biophys. Acta Gen. Subj..

[43] Morabito M.A., Llewellyn L.E., Moczydlowski E.G. (1995). Expression of saxiphilin in insect cells and localization of the saxitoxin-binding site to the *C*-terminal domain homologous to the C-lobe of transferrins. Biochemistry.

[44] Llewellyn L.E., Moczydlowski E.G. (1994). Characterization of saxitoxin binding to saxiphilin, a relative of the transferrin family that displays pH-dependent ligand-binding. Biochemistry.

[45] Cusick K.D., Minkin S.C., Dodani S.C., Chang C.J., Wilhelm S.W., Sayler G.S. (2012). Inhibition of copper uptake in yeast reveals the copper transporter Ctr1p as a potential molecular target of saxitoxin. Environ. Sci. Technol..

[46] Hill K., Hassett R., Kosman D., Merchant S. (1996). Regulated copper uptake in *Chlamydomonas reinhardtii* in response to copper availabilty. Plant. Physiol..

[47] Dancis A., Haile D., Yuan D.S., Klausner R.D. (1994). The *Saccharomyces cerevisiae* copper transport protein (Ctr1p). Biochemical characterization, regulation by copper, and physiologic role in copper uptake. J. Biol. Chem..

[48] Dancis A., Yuan D.S., Haile D., Askwith C., Eide D., Moehle C., Kaplan J., Klausner R.D. (1994). Molecular characterization of a copper transport protein in *S.cerevisiae*: An unexpected role for copper in iron transport. Cell.

[49] Page M.D., Kropat J., Hamel P.P., Merchant S.S. (2009). Two *Chlamydomonas* CTR copper transporters with a novel Cys-Met motif are localized to the plasma membrane and function in copper assimilation. Plant Cell.

[50] Aller S.G., Unger V.M. (2006). Projection structure of the human copper transporter CTR1 at 6-Ã resolution reveals a compact trimer with a novel channel-like architecture. Proc. Natl. Acad. Sci. USA.

[51] Henderson R., Ritchie J.M., Strichartz G.R. (1974). Evidence that tetrodotoxin and saxitoxin act at a metal cation binding site in sodium channels of nerve membrane. Proc. Natl. Acad. Sci. USA.

[52] De Feo C.J., Aller S.G., Unger V.M. (2007). A structural perspective on copper uptake in eukaryotes. Biometals.

[53] Shimizu Y. (1996). Microalgal metabolites: A new perspective. Annu. Rev. Microbiol..

[54] Kellmann R., Mihali T.K., Jeon Y.J., Pickford R., Pomati F., Neilan B.A. (2008). Biosynthetic intermediate analysis and functional homology reveal a saxitoxin gene cluster in cyanobacteria. App. Environ. Microbiol..

[55] Kellmann R., Neilan B.A. (2007). Biochemical characterization of paralytic shellfish toxin biosynthesis *in vitro*. J. Phycol..

[56] Mihali T.K., Kellmann R., Neilan B.A. (2009). Characterisation of the paralytic shellfish toxin biosynthesis gene clusters in *Anabaena circinalis* AWQC131C and *Aphanizomenon* sp. NH-5. BMC Biochem..

[57] Mihali T.K., Carmichael W.W., Neilan B.A. (2011). A putative gene cluster from a *Lyngbya wollei* bloom that encodes paralytic shellfish toxin biosynthesis. PLoS One.

[58] Lin S.J. (2011). Genomic understanding of dinoflagellates. Res. Microbiol..

[59] Moustafa A., Evans A.N., Kulis D.M., Hackett J.D., Erdner D.L., Anderson D.M., Bhattacharya D. (2010). Transcriptome profiling of a toxic dinoflagellate reveals a gene-rich protist and a potential impact on gene expression due to bacterial presence. PLoS One.

[60] Hou Y., Lin S. (2009). Distinct gene number-genome size relationships for eukaryotes and non-eukaryotes: Gene content estimation for dinoflagellate genomes. PLoSOne.

[61] Erdner D.L., Anderson D.M. (2006). Global transcriptional profiling of the toxic dinoflagellate *Alexandrium fundyense* using massively parallel signature sequencing. BMC Genomics.

[62] Stuken A., Orr R.J.S., Kellmann R., Murray S.A., Neilan B.A., Jakobsen K.S. (2011). Discovery of nuclear-encoded genes for the neurotoxin saxitoxin in dinoflagellates. PLoS One.

[63] Hackett J.D., Wisecarver J.H., Brosnahan M.L., Kulis D.M., Anderson D.M., Bhattacharya D., Plumley F.G., Erdner D.L. (2013). Evolution of saxitoxin synthesis in cyanobacteria and dinoflagellates. Mol. Biol. Evol..

[64] Bachvaroff T.R., Place A.R. (2008). From stop to start: Tandem gene arrangement, copy number and trans-splicing sites in the dinoflagellate *Amphidinium carterae*. PLoS One.

[65] Le Q.H., Markovic P., Hastings J.W., Jovine R.V.M., Morse D. (1997). Structure and organization of the peridinin chlorophyll a binding protein gene in *Gonyaulax polyedra*. Mol. Gen. Genet..

[66] Moustafa A., Loram J.E., Hackett J.D., Anderson D.M., Plumley F.G., Bhattacharya D. (2009). Origin of saxitoxin biosynthetic genes in cyanobacteria. PLoS One.

[67] Salcedo T., Upadhyay R.J., Nagasaki K., Bhattacharya D. (2012). Dozens of toxin-related genes are expressed in a nontoxic strain of the dinoflagellate *Heterocapsa circularisquama*. Mol. Biol. Evol..

[68] Yoshida T., Sako Y., Uchida A., Kakutani T., Arakawa O., Noguchi T., Ishida Y. (2002). Purification and characterization of sulfotransferase specific to O-22 of 11-hydroxy saxitoxin from the toxic dinoflagellate *Gymnodinium catenatum* (Dinophyceae). Fish. Sci..

[69] Sako Y., Yoshida T., Uchida A., Arakawa O., Noguchi T., Ishida Y. (2001). Purification and characterization of a sulfotransferase specific to N-21 of saxitoxin and gonyautoxin 2 + 3 from the toxic dinoflagellate *Gymnodinium catenatum* (Dinophyceae). J. Phycol..

[70] Landsberg J.H., Hall S., Johannessen J.N., White K.D., Conrad S.M., Abbott J.P., Flewelling L.J., Richardson R.W., Dickey R.W., Jester E.L.E. (2006). Saxitoxin puffer fish poisoning in the United States, with the first report of *Pyrodinium bahamense* as the putative toxin source. Environ. Health Perspect..

[71] Etheridge S.M. (2010). Paralytic shellfish poisoning: Seafood safety and human health perspectives. Toxicon.

[72] Deeds J.R., Landsberg J.H., Etheridge S.M., Pitcher G.C., Longan S.W. (2008). Non-traditional vectors for paralytic shellfish poisoning. Mar. Drugs.

[73] Van Dolah F.M. (2000). Marine algal toxins: Origins, health effects, and their increased occurrence. Environ. Health Perspect..

[74] Landsberg J.H. (2002). The effects of harmful algal blooms on aquatic organisms. Rev. Fish. Sci..

[75] Abbott J.P., Flewelling L.J., Landsberg J.H. (2009). Saxitoxin monitoring in three species of Florida puffer fish. Harmful Algae.

[76] Tamplin M.L., Hall S.L., Strichartz G. (1990). A Bacterial Source of Tetrodotoxins and Saxitoxins. Marine Toxins: Origin, Structure, and Molecular Pharmacology.

[77] Schantz E.J., McFarren E.F., Schafer M.L., Lewis K.H. (1958). Purified shellfsih poison for bioassay standardization. J. Assoc. Off. Anal. Chem..

[78] Aune T., Ramstad H., Heidenreich B., Landsverk T., Waaler T., Egaas E., Julshamn K. (1998). Zinc accumulation in oysters giving mouse deaths in paralytic shellfish poisoning bioassay. J. Shellfish Res..

[79] Turner A.D., Dhanji-Rapkova M., Algoet M., Suarez-Isla B.A., Cordova M., Caceres C., Murphy C.J., Casey M., Lees D.N. (2012). Investigations into matrix components affecting the performance of the official bioassay reference method for quantitation of paralytic shellfish poisoning toxins in oysters. Toxicon.

[80] Aune T., Aasen J.A.B., Miles C.O., Larsen S. (2008). Effect of mouse strain and gender on LD50 of yessotoxin. Toxicon.

[81] Food and Agricultural Organization (2004). Marine Biotoxins FAO Food and Nutrition Paper 80.

[82] Humpage A.R., Magalhaes V.F., Froscio S.M. (2010). Comparison of analytical tools and biological assays for detection of paralytic shellfish poisoning toxins. Anal. Bioanal. Chem..

[83] Louzao M.C., Vieytes M.R., Baptista de Sousa J.M.V., Leira F., Botana L.M. (2001). A fluorometric method based on changes in membrane potential for screening paralytic shellfish toxins in mussels. Anal. Biochem..

[84] Cheng J.P., Pi S.S., Ye S.F., Gao H.M., Yao L., Jiang Z.Y., Song Y.L., Xi L. (2012). A new simple screening method for the detection of paralytic shellfish poisoning toxins. Chin. J. Ocean. Limnol..

[85] Sullivan J.J., Hall S., Strichartz G. (1990). High-Performance Liquid Chromatographic Method Applied to Paralytic Shellfish Poisoning Research. Marine Toxins: Origin, Structure, and Molecular Pharmacology.

[86] Turner A.D., Hatfield R.C. (2012). Refinement of AOAC official method (SM) 2005.06 liquid chromatography-fluorescence detection method to improve performance characteristics for the determination of paralytic shellfish toxins in king and queen scallops. J. AOAC Int..

[87] Dell’Aversano C., Eaglesham G.K., Quilliam M.A. (2004). Analysis of cyanobacterial toxins by hydrophilic interaction liquid chromatography-mass spectrometry. J. Chromatogr. A.

[88] Dell’Aversano C., Hess P., Quilliam M.A. (2005). Hydrophilic interaction liquid chromatography-mass spectrometry for the analysis of paralytic shellfish poisoning (PSP) toxins. J. Chromatogr. A.

[89] Halme M., Rapinoja M.L., Karjalainen M., Vanninen P. (2012). Verification and quantification of saxitoxin from algal samples using fast and validated hydrophilic interaction liquid chromatography-tandem mass spectrometry method. J. Chromatogr. B.

[90] Davio S.R., Fontelo P.A. (1984). A competitive displacement assay to detect saxitoxin and tetrodotoxin. Anal. Biochem..

[91] Vieytes M.R., Cabado A.G., Alfonso A., Louzao M.C., Botana A.M., Botana L.M. (1993). Solid-phase radioreceptor assay for paralytic shellfish toxins. Anal. Biochem..

[92] Doucette G.J., Logan M.M., Ramsdell J.S., van Dolah F.M. (1997). Development and preliminary validation of a microtiter plate-based receptor binding assay for paralytic shellfish poisoning toxins. Toxicon.

[93] Usup G., Leaw C.-P., Cheah M.-Y., Ahmad A., Ng B.-K. (2004). Analysis of paralytic shellfish poisoning congeners by a sodium channel receptor binding assay. Toxicon.

[94] Van Dolan F.M., Fire S.E., Leighfield T.A., Mikulski C.M., Doucette G.J. (2012). Determination of paralytic shellfish toxins in shellfish by Receptor Binding Assay: Collaborative study. J. AOAC Int..

[95] Kogure K., Tamplin M.L., Simidu U., Colwell R.R. (1988). A tissue culture assay for tetrodotoxin, saxitoxin, and related toxins. Toxicon.

[96] Jellett J.F., Marks L.J., Stewart J.E., Dorey M.I., Watson-Wright W., Lawrence J.F. (1992). Paralytic shellfish poison (saxitoxin family) bioassays: Automated endpoint determination and stadardization of the *in vitro* tissue culture bioassay, and comparison with the standard mouse bioassay. Toxicon.

[97] Gallacher S., Birkbeck T.H. (1992). A tissue culture assay for direct detection of sodium channel blocking toxins in bacterial culture supernatents. FEMS Microbiol. Lett..

[98] Manger M.L., Lega L.S., Lee S.Y., Hungerford J.M., Wekell M.M. (1993). Tetrazolium-based bioassay for neurotoxins active on voltage-sensitive sodium channels: Semiautomated assay for saxitoxins, brevetoxins, and ciguatoxins. Anal. Biochem..

[99] Jellett J.F., Doucette L.I., Belland E.R. (1998). The MIST (TM) shipable cell bioassay kits for PSP: An alternative to the mouse bioassay. J. Shellfish Res..

[100] Nicholson R.A., Li G.H., Buenaventura E., Graham D. (2002). A rapid and sensitive assay for paralytic shellfish poison (PSP) toxins using mouse brain synaptoneurosomes. Toxicon.

[101] Louzao M.C., Vieytes M.R., Cabado A.G., de Sousa J., Botana L.M. (2003). A fluorimetric microplate assay for detection and quantitation of toxins causing paralytic shellfish poisoning. Chem. Res. Toxicol..

[102] Manger R., Woodle D., Berger A., Hungerford J. (2007). Flow cytometric detection of saxitoxins using fluorescent voltage-sensitive dyes. Anal. Biochem..

[103] Usleber E., Schneider E., Terplan G. (1991). Direct enzyme immunoassay in microtitration plate and test strip format for the detection of saxitoxin in shellfish. Lett. Appl. Microbiol..

[104] Chu F.S., Huang X., Hall S. (1992). Production and characterization of antibodies against neosaxitoxin. J. AOAC Int..

[105] Burk C., Usleber E., Dietrich R., Martlbauer E. (1995). Production and characterization of antibodies against neosaxitoxin utilizing a novel immunogen synthesis procedure. Food Agric. Immunol..

[106] Micheli L., di Stefano S., Moscone D., Palleschi G., Marini S., Coletta M., Draisci R., Quadri F.D. (2002). Production of antibodies and development of highly sensitive formats of enzyme immunoassay for saxitoxin analysis. Anal. Bioanal. Chem..

[107] Huang X., Hsu K.H., Chu F.S. (1996). Direct competitive enzyme-linked immunosorbent assay for saxitoxin and neosaxitoxin. J. Agric. Food Chem..

[108] Kawatsu K., Hamano Y., Sugiyama A., Hashizume K., Noguchi T. (2002). Development and application of an enzyme immunoassay based on a monoclonal antibody against gonyautoxin components of paralytic shellfish poisoning toxins. J. Food Prot..

[109] Dubois M., Demoulin L., Charlier C., Singh G., Godefroy S.B., Campbell K., Elliott C.T., Delahaut P. (2010). Development of ELISAs for detecting domoic acid, okadaic acid, and saxitoxin and their applicability for the detection of marine toxins in samples collected in Belgium. Food Addit. Contam. Part A.

[110] Garneau M.E., Schnetzer A., Countway P.D., Jones A.C., Seubert E.L., Caron D.A. (2011). Examination of the seasonal dynamics of the toxic dinoflagellate *Alexandrium catenella* at Redondo Beach, California, by quantitative PCR. Appl. Environ. Microbiol..

[111] Murray S.A., Wiese M., Stuken A., Brett S., Kellmann R., Hallegraeff G., Neilan B.A. (2011). *SxtA*-based quantitative molecular assay to identify saxitoxin-producing harmful algal blooms in marine waters. Appl. Environ. Microbiol..

[112] Al-Tebrineh J., Pearson L.A., Yasar S.A., Neilan B.A. (2012). A multiplex qPCR targeting hepato- and neurotoxigenic cyanobacteria of global significance. Harmful Algae.

[113] Nagai S., Itakura S. (2012). Specific detection of the toxic dinoflagellates *Alexandrium tamarense* and *Alexandrium catenella* from single vegetative cells by a loop-mediated isothermal amplification method. Mar. Genomics.

[114] Notomi T., Okayama H., Masubuchi H., Yonezawa T., Watanabe K., Amino N., Hase T. (2000). Loop-mediated isothermal amplification of DNA. Nucl. Acids Res..

[115] Mori Y., Nagamine K., Tomita N., Notomi T. (2001). Detection of loop-mediated isothermal amplification reaction by turbidity derived from magnesium pyrophosphate formation. Biochem. Biophys. Res. Commun..

[116] Enosawa M., Kageyama S., Sawai K., Watanabe K., Notomi T., Onoe S., Mori Y., Yokomizo Y. (2003). Use of loop-mediated isothermal amplification of the IS900 sequence for rapid detection of cultured *Mycobacterium avium* subsp. *paratuberculosi*s. J. Clin. Microbiol..

[117] Tang X.H., Yu R.C., Zhou M.J., Yu Z.G. (2012). Application of rRNA probes and fluorescence *in situ* hybridization for rapid detection of the toxic dinoflagellate *Alexandrium minutum*. Chin. J. Ocean. Limnol..

[118] Rhodes L., Smith K., De Salas M. (2007). DNA probes, targeting large sub-unit rRNA, for the rapid identification of the paralytic shellfish poison producing dinoflagellate, *Gymnodinium catenatum*. New Z. J. Mar. Freshw. Res..

[119] Wyatt T., Jenkinson I.R. (1997). Notes on *Alexandrium* population dynamics. J. Plankton Res..

[120] Ritson-Williams R., Yotsu-Yamashita M., Paul V.J. (2006). Ecological functions of tetrodotoxin in a deadly polyclad flatworm. Proc. Natl. Acad. Sci. USA.

[121] Cembella A.D. (2003). Chemical ecology of eukaryotic microalgae in marine ecosystems. Phycologia.

[122] Anderson D.M., Cheng T.P.-O. (1988). Intracellular localization of saxitoxins in the dinofllagellate *Gonyaulax tamarensis*. J. Phycol..

[123] Cembella A.D., Anderson D.M., Cembella A.D., Hallegraeff G. (1998). Ecophysiology and Metabolism of Paralytic Shellfish Toxins in Marine Microalgae. Physiological Ecology of Harmful Algal Blooms.

[124] Zimmer R.K., Ferrer R.P. (2007). Neuroecology, chemical defense, and the keystone species concept. Biol. Bull..

[125] Oikawa H., Satomi M., Watabe S., Yano Y. (2005). Accumulation and depuration rates of paralytic shellfish poisoning toxins in the shore crab *Telmessus acutidens* by feeding toxic mussels under laboratory controlled conditions. Toxicon.

[126] Robineau B., Gagné J.A., Fortier L., Cembella A.D. (1991). Potential impact of a toxic dinoflagellate (*Alexandrium excavatum*) bloom on survival of fish and crustacean larvae. Mar. Biol..

[127] Selander E., Thor P., Toth G., Pavia H. (2006). Copepods induce paralytic shellfish toxin production in marine dinoflagellates. Proc. R. Soc. B.

[128] Bergkvist J., Selander E., Pavia H. (2008). Induction of toxin production in dinoflagellates: The grazer makes a difference. Oecologia.

[129] Teegarden G.J. (1999). Copepod grazing selection and particle discrimination on the basis of PSP toxin content. Mar. Ecol. Prog. Ser..

[130] Da Costa R.M., Franco J., Cacho E., Fernandez F. (2005). Toxin content and toxic effects of the dinoflagellate *Gyrodinium corsicum* (Paulmier) on the ingestion and survival rates of the copepods *Acartia grani* and *Euterpina acutifrons*. J. Exp. Mar. Biol. Ecol..

[131] Frangoulos M., Guisande C., Maneiro I., Riveiro I., Franco J. (2000). Short-term and long-term effects of the toxic dinoflagellate *Alexandrium minutum* on the copepod *Acartia clausi*. Mar. Ecol. Prog. Ser..

[132] Barreiro A., Guisande C., Frangopulos M., Gonzalez-Fernandez A., Munoz S., Perez D., Magadan S., Maneiro I., Riveiro I., Iglesias P. (2006). Feeding strategies of the copepod *Acartia clausi* on single and mixed diets of toxic and non-toxic strains of the dinoflagellate *Alexandrium minutum*. Mar. Ecol. Prog. Ser..

[133] Turner J.T., Tester P.A. (1997). Toxic marine phytoplankton, zooplankton grazers, and pelagic food webs. Limnol. Oceanogr..

[134] Solé J., Estrada M., Garcia-Ladona E. (2006). Biological control of harmful algal blooms: A modelling study. J. Mar. Syst..

[135] Tillmann U., John U. (2006). Toxic effects of *Alexandrium* spp. on heterotrophic dinoflagellates: An allelochemical defence mechanism independent of PSP-toxin content. Mar. Ecol. Prog. Ser..

[136] Juhl A.R., Martins C.A., Anderson D.M. (2008). Toxicity of *Alexandrium lusitanicum* to gastropod larvae is not caused by paralytic-shellfish-poisoning toxins. Harmful Algae.

[137] Ianora A., Bentley M.G., Caldwell G.S., Casotti R., Cembella A.D., Engstrom-Ost J., Halsband C., Sonnenschein E., Legrand C., Llewellyn C.A. (2011). The relevance of marine chemical ecology to plankton and ecosystem function: An emerging field. Mar. Drugs.

